# Audit Reviewing Consent for Electro Convulsive Therapy

**DOI:** 10.1192/bjo.2022.271

**Published:** 2022-06-20

**Authors:** Saba Ansari, Sujatha Maiya

**Affiliations:** 1 University Hospital Monklands, Airdrie, United Kingdom; 2 University Hospital Wishaw, Wishaw, United Kingdom

## Abstract

**Aims:**

The National Institute for Health and Clinical Excellence (NICE) recommends that valid consent be sought for Electro Convulsive Therapy (ECT) in all instances where the individual has the capacity to provide or deny consent. Individuals should get comprehensive information on the general risks and potential advantages of ECT. When informed consent and decision-making are not possible, advance directives are fully considered, and the individual's advocate and caregiver are consulted. Additionally, patients should be informed that they can discontinue treatment at any moment. The purpose of this audit is to determine whether we are adhering to the NICE-recommended standards and recommendations.

**Methods:**

This is a retrospective audit looking at case notes from the last 30 individuals who received ECT at University Hospital Wishaw. Individuals' electronic and paper light notes were analysed for data.

In informal patients, the aspects reviewed were:

1. Documentation about adequate information given.
2. Documentation of risks and benefits explained.
3. Documentation of information given about withdrawing consent.

In Formal patients the aspects reviewed were:

1. Number of cases who received urgent ECT under Mental Health Act (Scotland) with Record of notification on T4 form.
2. Number of cases who received ECT under Mental Health Act (Scotland) with Certificate of the designated medical practitioner completing T3A form.
3. Number of cases who regained capacity to consent for ECT during the course of treatment and had appropriate informed consent with Certificate of consent to treatment completed on T2 form.
4. Did any of the cases have Advance Statement either for or against having ECT as a treatment option for them?

**Results:**

Observations of the data collected revealed that over 30% of cases lacked the documentation proposed by NICE standards. Only 25% of cases with complete documentation were informal patients, whereas the remaining 75% received ECT under the Mental Health Act Scotland.

**Conclusion:**

Based on the observations, this audit establishes that our results do not meet generally accepted standards. The full results will be disseminated with appropriate recommendations to the prescribing Consultant Psychiatrists. This Audit process has also prompted us to redesign the ECT booklet to include the required documents in accordance with standards.

